# Whole Body Mechanics of Stealthy Walking in Cats

**DOI:** 10.1371/journal.pone.0003808

**Published:** 2008-11-26

**Authors:** Kristin L. Bishop, Anita K. Pai, Daniel Schmitt

**Affiliations:** 1 Section of Ecology and Evolution, University of California Davis, Davis, California, United States of America; 2 Department of Biological Anthropology and Anatomy, Duke University, Durham, North Carolina, United States of America; Harvard University, United States of America

## Abstract

The metabolic cost associated with locomotion represents a significant part of an animal's metabolic energy budget. Therefore understanding the ways in which animals manage the energy required for locomotion by controlling muscular effort is critical to understanding limb design and the evolution of locomotor behavior. The assumption that energetic economy is the most important target of natural selection underlies many analyses of steady animal locomotion, leading to the prediction that animals will choose gaits and postures that maximize energetic efficiency. Many quadrupedal animals, particularly those that specialize in long distance steady locomotion, do in fact reduce the muscular contribution required for walking by adopting pendulum-like center of mass movements that facilitate exchange between kinetic energy (KE) and potential energy (PE) [1]–[4]. However, animals that are not specialized for long distance steady locomotion may face a more complex set of requirements, some of which may conflict with the efficient exchange of mechanical energy. For example, the “stealthy” walking style of cats may demand slow movements performed with the center of mass close to the ground. Force plate and video data show that domestic cats (*Felis catus,* Linnaeus, 1758) have lower mechanical energy recovery than mammals specialized for distance. A strong negative correlation was found between mechanical energy recovery and diagonality in the footfalls and there was also a negative correlation between limb compression and diagonality of footfalls such that more crouched postures tended to have greater diagonality. These data show a previously unrecognized mechanical relationship in which crouched postures are associated with changes in footfall pattern which are in turn related to reduced mechanical energy recovery. Low energy recovery was not associated with decreased vertical oscillations of the center of mass as theoretically predicted, but rather with posture and footfall pattern on the phase relationship between potential and kinetic energy. An important implication of these results is the possibility of a tradeoff between stealthy walking and economy of locomotion. This potential tradeoff highlights the complex and conflicting pressures that may govern the locomotor choices that animals make.

## Introduction

It has long been known that animals that walk and run use energy-saving mechanisms that reduce the amount of muscular contribution required to accelerate and decelerate the center of mass during locomotion [1], [reviewed in 2]. Although widely accepted, most of the data supporting these models among vertebrates have been derived mainly from species that are specialized for steady locomotion over long distances, including humans, large birds, dogs, and horses [1]–[4]. It is often assumed that minimizing energetic costs during steady locomotion represents the most important selection pressure on the organ systems that support locomotion. This appears to be the case for long-distance specialists [1]–[4]. However, the extent to which mammals with other locomotor priorities use these energy saving mechanisms remains less well explored. Studies of monkeys [1], frogs [5], lizards [6], and tortoises [7] have shown greater variability in walking mechanics with respect to walking speed and lower mechanical energy recovery than is seen in distance specialists, yet little attention has been devoted to the reasons for these differences. Without such data it is difficult to evaluate the critical variables that influence gait and postural choices in animals.

One way to explore this problem is to consider the compromises that animals may have to make when balancing the value of locomotor behaviors that reduce muscular effort and those that promote other important performance targets. Cats represent an ideal model animal to explore the balance of different locomotor goals such as energetic efficiency and stealth because cats consistently walk with their limbs more flexed than distance specialists and because they often adopt a stealthy hunting style that requires approaching prey without being detected.

The ways in which the stealthy gait used by cats may reduce locomotor efficiency are clear from consideration of current models for locomotor mechanics. The energy saving mechanism that is thought to be used during walking is a pendulum-like mechanical energy exchange in which kinetic energy (KE) is stored temporarily as gravitational potential energy (PE) as the center of mass rises and vaults over the relatively stiff stance leg, and is then returned as KE as the animal falls under gravity [1]–[5]. If PE and KE fluctuations are precisely out of phase and of the same magnitude, then the total external mechanical energy of the system remains constant and no energetic input is required to sustain motion.

The exchange of potential and kinetic energy reduces the muscular contribution needed to accelerate and decelerate the center of mass, although there is currently little data showing a direct relationship between mechanical energy exchange and metabolic costs. In some animals, especially small ones, the savings may be relatively low. However, the amount of muscular effort needed to control movements of the center of mass is reduced as exchange increases. As a result, the costs of locomotion are reduced by some percentage as energetic exchange increases. This mechanism has been well-documented in animals that habitually use steady, relatively long-distance locomotor strategies; for example, dogs can recover up to 70% of the mechanical energy used while walking [3], [8]. Tortoises and frogs in contrast have low levels of energetic exchange [5], [7]. There is only one previous study [9] that explored center of mass movements in cats, and that study included only one step in the analysis. Cats, which use a broad range from more to less crouched postures (Video S1, Video S2), provide an ideal opportunity to parse the specific relationships between footfall patterns and limb posture and energetic exchange in mammals.

## Results and Discussion

The degree to which potential and kinetic energy can be effectively exchanged in an inverted pendulum model depends on both the relative magnitudes of PE and KE fluctuations and their phase relationship. Potential energy fluctuations can be influenced by the amount of vertical displacement of the center of mass, which in turn can be influenced by the amount of limb flexion during stance phase. Increased limb flexion decreases the effective limb length and may reduce the vertical oscillation of the center of mass if other variables such as limb protraction and angular excursion remain equal. Therefore, we hypothesized that because cats walk with greater limb flexion than distance specialists, they may have lower mechanical energy recovery compared to long distance specialists because of reduced oscillations in the vertical position of their center of mass if the other variables that affect recovery such as amplitude of KE fluctuations and phase relationship between PE and KE remain unchanged. Accounting for limb compression is important for a full understanding of walking mechanics in animals. A recent study modeling human walking found that a pendulum model with completely stiff limbs that produced reasonable predictions of percent recovery did a poor job of predicting the force profile of actual human walking and that incorporating spring elements due to limb compression (flexion) produced a much closer match with empirical data [10].

**Figure 1:**
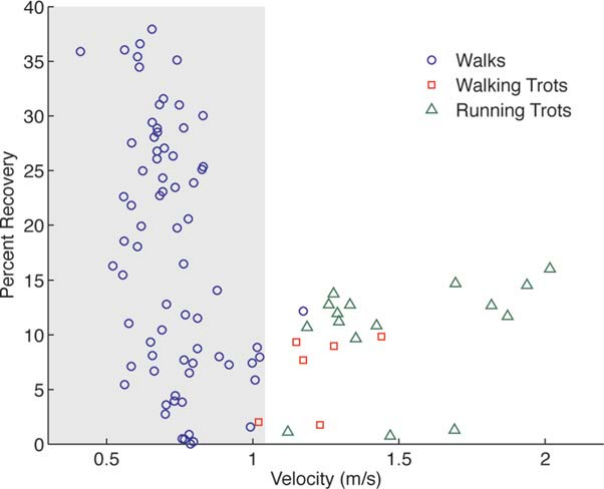
Percent recovery of mechanical energy due to exchange of PE and KE *vs.* velocity for walking and trotting strides. Shaded area delineates the range of typical walking speeds. The walk/trot transition occurs around 1 m/s, which corresponds to a Froude number of 0.5 for these cats. Recovery values all fall below 40% and there is a low correlation between energy recovery and velocity for walking strides (linear fit: r^2^ = 0.1779, p = 0.002; quadratic fit: r^2^ = 0.1830, p = 0.002).

Our data support the hypothesis that cats have a less effective pendular mechanical energy-saving mechanism than distance specialists. The average energy recovery (17.6±11.3% (s.d.), n = 6) achieved by the cats was very low and their maximum energy recovery (37.9%) was substantially lower than the maximum energy recovery reported for distance specialists [1], [3]. Energy recovery values for trots were all relatively low suggesting a clear walk-trot transition (Fig. 1). Considering only walks, there was a statistically significant regression slope, but very poor predictive power in the relationship between velocity and recovery (linear fit: r^2^ = 0.1779, p = 0.002; quadratic fit: r^2^ = 0.1830, p = 0.002, n = 6). Although previous studies on walking mechanics [1], [3] found a hyperbolic, curvilinear relationship between velocity and percent recovery with optimal mechanical energy recovery at a moderate speed in a small number of distance specialists, the cats in this study showed no evidence of such a relationship (Figure 1). This suggests that velocity is not as good a predictor of mechanical energy recovery in cats as it has been found to be in distance specialists and other explanations must be sought for variation in recovery. Similar variability in energy recovery with respect to velocity has been documented in reptiles and amphibians [5]–[7], [11] and uniformly low energy recovery across a range of speeds was found in small mammals [12], [13]. It appears therefore that animals that do not specialize in long distance travel show a much less stereotyped pattern than that of distance specialists. However, previous studies did not explore the source of this variation.

The timing of footfalls also can affect the magnitude of PE fluctuations. Diagonality is defined as the percentage of a stride by which ipsilateral (same side) feet follow one another. In a pacing gait, the ipsilateral front and hind feet strike the ground at the same time and the diagonality is 0%. In a trot, the ipsilateral front and hind feet strike the ground half the stride time apart, and the diagonality is 50%. For a stride with a diagonality of 25%, the contacts of the four feet are evenly spaced in time. It has been argued on theoretical grounds that if mass is evenly distributed between the forelimbs and hind limbs and an animal walks with a diagonality of 25%, the vertical position of its center of mass will move so little as to allow almost no energy exchange [3]. This is because at the point in the stride when the front end is at its highest point, the hind end is at its lowest point and vice versa, so the front and back ends oscillate around the center of mass, resulting in zero PE fluctuation [3]. It has been shown [3] that dogs avoid this problem both by having more of their mass concentrated anteriorly and by consistently using a diagonality around 15% when walking.

**Figure 2:**
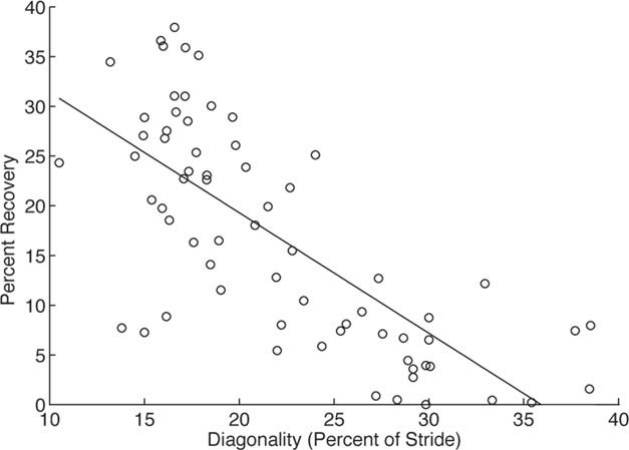
Percent recovery of mechanical energy due to exchange of PE and KE *vs.* diagonality for walking strides. Diagonality is defined as the percentage of a stride by which ipsilateral (same side) feet follow one another. There is a strong negative correlation between recovery and diagonality such that energy recovery decreases as the footfalls becomes more evenly spaced in time (linear fit: r^2^ = 0.5417, p<0.001; quadratic fit: r^2^ = 0.5567, p<0.001).

Griffin et al.'s [3] argument that dogs maintained high levels of recovery because of the mass distribution bias toward the forelimbs and because dogs chose diagonalities close to 15%, and that lower or higher diagonalities would lower mechanical energy recovery, is a logical conclusion but has not been fully tested because the dogs in this study used a narrow range of diagonalities. Cats are an ideal model in which to study the effect of diagonality on recovery. Like dogs, cats have a weight distribution pattern biased toward the forelimb [14]. However, cats naturally use a wide range of diagonalities while walking (Fig. 2). Therefore the data presented here can be used to directly test Griffin et al.'s [3] theoretically predicted relationship between footfall pattern and pendular energy recovery.

The prediction that diagonality influences mechanical energy recovery is supported by our results. Cats in this study often used footfall patterns with diagonality close to 25% and had low energy recovery when they did. In walking gaits, energy recovery was significantly, inversely correlated with diagonality (linear fit: r^2^ = 0.5417, p<0.001, n = 5) (Fig. 2). This supports Griffin's [3] model and is consistent with other studies that found recoveries that were low and/or highly variable in animals that also used gaits with a high diagonality [5]–[7], [11], [12].

A second important factor that can influence mechanical energy recovery is the phase relationship between PE and KE fluctuations. Phase relationships are typically computed by identifying peaks in the energy fluctuations, but clear energy peaks are often not readily apparent. In these cases the phase relationship can be characterized by calculating congruity, the product of the derivatives of PE and KE with respect to time [5]. Congruity is positive when PE and KE change in the same direction and negative when they change in opposite directions. This can be expressed as a percentage of the stride during which congruity is positive. A high percent congruity indicates that PE and KE fluctuate largely in phase allowing little mechanical energy recovery, whereas a low percent congruity is associated with out of phase PE and KE fluctuations and high energy recovery.

**Figure 3:**
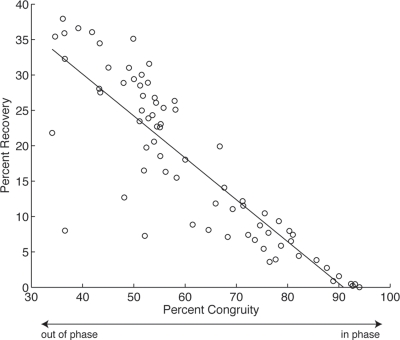
Percent recovery of mechanical energy due to exchange of PE and KE *vs.* percent congruity for walking strides. The majority of the variation in recovery is explained by variation in congruity, indicating that the phase relationship between changes in PE and KE is the primary determinant of mechanical energy recovery (linear fit: r^2^ = 0.7351, p≪0.001; quadratic fit: r^2^ = 0.7352, p≪0.001).

The cats in this study had a strong, significant relationship (r^2^ = 0.7351, p≪0.001, n = 6) between recovery and congruity, thus changes in phase of PE and KE fluctuations explain the vast majority of the variation in recovery (Fig. 3). This contrasts with our prediction that the magnitude of vertical oscillations of the center of mass would be the primary influence on mechanical energy recovery; our data reject this hypothesis. No relationship was found between energy recovery and the maximum vertical displacement of the center of mass during a stride (linear fit r^2^ = 0.0480, p = 0.0607, n = 6). Therefore, diagonality appears to influence energy recovery not through its effect on vertical oscillations of the center of mass as theoretically predicted, but through an effect on the phase relationship between PE and KE fluctuations.

**Figure 4:**
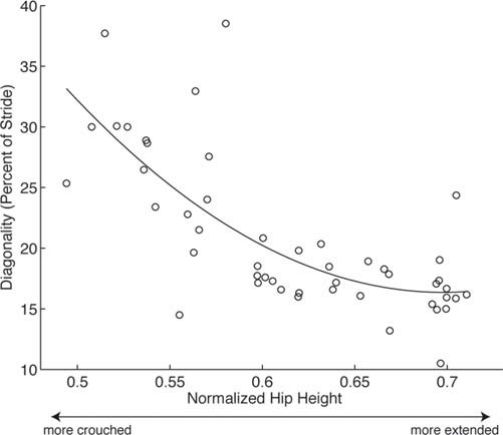
Diagonality *vs.* mean normalized hip height during stance phase for walking strides. The height of the hip was normalized by total leg length. There is a strong negative correlation between diagonality and hip height such that footfall pattern becomes more evenly spaced when limbs are more compressed (linear fit: r^2^ = 0.4959, p<0.001; quadratic fit: r^2^ = 0.5455, p<0.001).

In this study, there was also a clear association between crouched posture and diagonality. The cats in our study used a wide range of postures from very crouched (Video S1) to relatively extended (Video S2), and the adoption of these different postures was not associated with walking speed. We found a significant curvilinear relationship between diagonality and normalized hip height (quadratic fit: r^2^ = 0.5455, p<0.001, n = 3) (Fig. 4). During crouched strides in which the hip was held relatively close to the substrate, cats used higher diagonalities. It is likely that evenly spaced footfalls increase stability [15] and are thus helpful during stealthy approach to prey. However, by choosing such diagonalities during crouched gaits, cats sacrifice energetic efficiency. The reduced mechanical energy recovery that results from this footfall pattern may be compensated somewhat by decreased collisional energy losses. In a numerical simulation of stiff-legged walking [8], it was found that collisional energy loss due to the change in direction of the center of mass was lowest when limb phase (diagonality) was 25%.

The data presented here suggest ways in which animals can balance complex and sometimes conflicting performance criteria when walking. Some animals may gain the greatest selective advantage by reducing metabolic energy costs by reducing muscular contribution whereas others gain the greatest advantage by avoiding detection while avoiding predators or stalking prey. Those animals may be unable to achieve high levels of mechanical energy recovery as a result of choosing more diagonal footfall patterns and more crouched postures which tend to increase the congruity of PE and KE fluctuations, but may gain stability during stealthy walking by choosing this kind of gait and posture. This novel study of cats highlights these compromises very clearly. The strong associations found between posture and diagonality, and between diagonality and mechanical energy recovery within this single species demonstrates that cats tune their behavior to different critical demands depending on the locomotor context. Cats provide a good comparison with distance specialists such as canids because they consistently walk with their limbs in a more flexed posture, which as we have seen here within cats, is associated with poorer mechanical energy exchange through its effect on footfall pattern. Dogs tend to use lower diagonalities and have higher energy recovery [3] than cats and it seems likely that this is due at least in part to their more extended posture. Little is yet known about the relationship between pendular energy recovery and the metabolic costs associated with walking, particularly in the more poorly studied non-distance specialists. If fluctuations in PE and KE are small to begin with, the cost of supplying this energy through muscular work may be small. Although further study is needed regarding the metabolic costs of reduced pendular exchange during walking, the consistent differences between the results found here and those reported for distance specialists suggest the possibility of an evolutionary tradeoff between energetic efficiency and stealthy locomotion, highlighting the importance of the interplay of conflicting performance goals to evolutionary outcomes.

## Materials and Methods

### Animals and Equipment

Methods followed Duke University IACUC guidelines (IACUC # A365-04-12). Six adult cats were used in this study. Animals were studied in two phases separated by one year. Three cats were studied in each phase. In both phases the cats were allowed to walk freely along a 6-meter runway. A 1.75-meter section of the runway was instrumented with a force plate, which measured ground reaction forces in three dimensions. For the first three cats a single multi-component Kistler (9281B) force platform (Kistler Corporation, Amherst, New York) was placed below the central section. For the remaining three cats four Kistler force sensors were built directly into the central section of the runway. Both plates were calibrated with the same weights. All force plate data were collected with and processed by Kistler Bioware software.

Full strides for all cats were videorecorded using a camera placed perpendicular to the animal's path of motion. The first three cats were videorecorded at 60 Hz using a Panasonic WVD5000 (Panasonic, Seacaucus, NJ). Force plate and video records were coordinated using an Event Video Control Unit (Peak Performance, Englewood Colorado).

The remaining three of the cats were partially shaved and marked with contrasting, non-toxic paint to aid in kinematic analysis. These cats were videorecorded at 125 Hz using a Photron Fastcam Super10K (San Diego, CA) digital video camera. Force plate and video signals were coordinated using an electronic trigger to initiate simultaneous video and force plate data collection. The marked cats had markers placed as close to the centers of rotation of the joints as possible at the shoulder, elbow, wrist, hip, knee, ankle, and metatarsal-phalangeal joint.

After data collection, the synchronized video and force data were cropped to a single stride. Force data were cropped within Bioware and video was cropped using Virtualdub video software. The markers and the footfalls were digitized using DLT Dataviewer [16] within Matlab (MathWorks, Natick, MA). The resulting x, y coordinate data were used to compute joint angles, limb duty factor (the proportion of the stride during which each limb is in contact with the ground), and diagonality. The hip marker was used to compute average forward velocity. A line was fit through the x coordinates of the hip position through the stride and the slope of the line was used as the average forward velocity. Only steady velocity strides were used in the analysis. To assess this we computed the residuals from the linear fit of the position data. This gives an estimate of how well the raw data are characterized by a steady velocity; any acceleration will result in high residuals. We excluded strides with maximum residuals greater than 5%. This estimate is conservative because it incorporates both digitizing error and deviations from steady velocity.

### Energy Calculations

The force data were processed using Matlab. The accuracy of the force measurements was verified both by calibrating the plate before and after each day of data collection and by comparing the vertical component of the ground reaction force over the entire stride to the body weight of the animal, taken just after data collection. Only trials in which the vertical force fell within 20% of body weight were used for further analysis. The methods for calculating center of mass movements and mechanical energy exchange followed those described elsewhere [1], [3], [5]. Measured forces were divided by the cat's body mass to compute accelerations and the accelerations were integrated twice to calculate the velocity and displacement of the center of mass. The average forward velocity measured from the video was used as the integration constant for the forward component of the instantaneous velocity calculations. The calculated velocities were detrended prior to the second integration to compute position. The displacement values were normalized around zero by subtracting the mean from each data point.

Potential energy was calculated from the vertical component of the displacement of the center of mass as
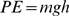
(1)where m is body mass, g is gravitational acceleration (9.8 ms^−2^), and h is the vertical position of the center of mass relative to its starting position. KE was calculated from the instantaneous velocity as
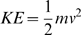
(2)where m is body mass and v is the magnitude of the resultant velocity vector. For each time step, total mechanical energy (TME) was calculated as the sum of PE and KE. Percent recovery was then computed as
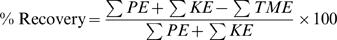
(3)where the sums refer to the sum of all positive energy changes over the course of the stride.

Several factors can influence the degree of mechanical energy recovery during a stride. The phase relationship between PE and KE peaks is a very important determinant of the amount of energy that can be exchanged. However, it is often the case, particularly in slow walks, that clear peaks in the energy profiles cannot be distinguished objectively [2], [5]. Following Ahn et al. [5], we avoid this problem by using a quantity called congruity. Congruity is computed as
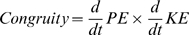
(4)


Congruity is therefore positive when PE and KE change in the same direction (in phase) and negative when they change in opposite directions (out of phase). As a summary value, we use percent congruity, defined as the percentage of the stride in which congruity is positive. A high percent congruity means that PE and KE fluctuate largely in phase and we expect low mechanical energy recovery, whereas a low percent congruity means that PE and KE fluctuate largely out of phase and we expect high energy recovery.

We quantified the extent to which cats adopted a crouched posture as a normalized hip height. This was calculated as the vertical component of the distance from the hip to the floor divided by the sum of the lengths of all of the leg segments, such that a normalized hip height of one means that the limb is fully extended and values below one approaching zero indicate greater limb flexion.

### Statistics

All correlations were computed using linear and quadratic least squares regression models with a significance level of p = 0.05. Results from regressions performed on the individuals separately did not conflict with those for the total data set. Only whole data set results are reported. Means are reported plus or minus one standard deviation.

## Supporting Information

Video S1 Crouched Walk. This cat is walking with its limbs highly flexed and its body low to the ground. This cat is walking at the same speed as the cat in the accompanying video who uses a more extended posture.
(0.33 MB MPG)

Video S2 Extended Walk. This cat is walking with its limbs relatively stiff and its body held high off the ground. This cat is walking at the same speed as the cat in the accompanying video who uses a more crouched posture.
(0.34 MB MPG)
